# Efficacy of Xinbao pill on chronic heart failure: Study protocol of a multicenter, randomized, double-blind, placebo-controlled trial

**DOI:** 10.3389/fphar.2022.1058799

**Published:** 2022-10-25

**Authors:** Yuanping Wang, Jiahua Li, Jiaqi Yan, Yulin Wang, Yuanyuan Cheng, Zhongqiu Liu, Dawei Wang

**Affiliations:** ^1^ Shunde Hospital of Guangzhou University of Chinese Medicine, Guangzhou University of Chinese Medicine, Guangzhou, Guangdong, China; ^2^ International Institute for Translational Chinese Medicine, Guangzhou University of Chinese Medicine, Guangzhou, Guangdong, China; ^3^ The First Clinical Medical College of Guangzhou University of Chinese Medicine, Guangzhou, Guangdong, China

**Keywords:** Xinbao pill, traditional chinese medicine, clinical trial, protocol, chronic heart failure

## Abstract

**Introduction:** Chronic heart failure (CHF) is a common cardiovascular disease. In China, Xinbao pill (XBP) is widely used as an adjuvant therapy for CHF. However, there is still a lack of high-quality clinical evidence. We designed this multicenter, randomized, double-blind, placebo-controlled trial to critically evaluate the efficacy and safety of XBP as an adjuvant treatment for patients with CHF.

**Methods and analysis:** We will recruit 284 patients with a clinical diagnosis of “heart-kidney yang deficiency syndrome” CHF receiving treatment in six hospitals in China. Patients will be randomly assigned, in a 1:1 ratio, to the treatment or control group using a central randomization system. All patients will receive conventional drug therapy for heart failure combined XBP (Guangdong Xinbao Pharmaceutical Co., Ltd., Guangdong, China) or a placebo. Study physicians, subjects, outcome assessors, and statisticians will be blinded to the group assignment. The primary outcome will be the change in the proportion of patients who show a decrease in serum NT-proBNP of more than 30% after treatment. Secondary outcomes are NYHA class, 6-minute walk distance test, Minnesota Quality of Life Scale score, endpoint events, serum NT-proBNP, echocardiographic parameters, and traditional Chinese medicine (TCM) symptom score. Adverse events will be monitored throughout the trial. Data will be analyzed according to a predetermined statistical analysis plan.

**Discussion:** The results of this study will provide solid evidence of the safety and efficacy of XBP as an alternative and complementary treatment measure for patients with CHF.

**Clinical Trial Registration:** Chinese Clinical Trial Registration Center (ChiCTR2000038492).

## Introduction

Chronic heart failure (CHF), an end-stage of various cardiac functional/organic diseases, is a major public health problem facing the world (Francis, 2001). Epidemiological data show that approximately 64.3 million people worldwide are afflicted by this disease (Groenewegen et al., 2020). In developed countries, the prevalence of heart failure is generally estimated at 1%–2% of the general adult population (Hao et al., 2019). As the population continues to increase in age, the prevalence of CHF is on the increasing, and it is estimated that by 2030, the prevalence of heart failure in the United States alone will increase by 46%, totaling more than eight million cases (Mozaffarian et al., 2016). In the United States, CHF is the leading cause of hospitalization for people older than 65 years of age, accounting for approximately one million hospitalizations each year (Rosamond et al., 2008), placing a tremendous burden on the individual and the health care system (Roger, 2021). The angiotensin-converting enzyme inhibitor (ACEI)/angiotensin receptor neprilysin inhibitor (ARNI), sodium glucose cotransporter two inhibitor, a beta-blocker, and mineralocorticoid receptor antagonists (MRA) are currently recommended in the guidelines as the basic therapy for CHF (McDonagh et al., 2021). Nevertheless, the prognosis of patients with CHF is not good. Studies have shown that the mortality rate of CHF patients within 1 month of discharge after receiving systemic and conventional basic treatment is still as high as approximately 10% (Pfeffer et al., 2019).

The Xinbao pill (XBP), a well-known Chinese patent medicine, was developed by Guangdong Xinbao Pharmaceutical Co., Ltd. (Guangdong, China) and approved by the China Food and Drug Administration (No: Z44021843) for the treatment of CHF. XBP is composed of nine herbal extracts (Table 1) and has been widely used in China in recent years for the treatment of cardiovascular diseases, especially for CHF. To explore the potential mechanism of XBP in the treatment of CHF, researchers have conducted a meaningful exploration. Previous studies have shown that in a rat model of CHF induced by abdominal aortic constriction surgery, XBP ameliorated the pathological changes of myocardial hypertrophy, disturbed cardiomyocyte arrangement and gap widening in CHF rats, and enhanced cardiac function (He et al., 2020). The mechanism might be related to the inhibition of phosphorylation activation of PI3K/Akt signaling and inhibition of GSK3β phosphorylation (He et al., 2020). It has also been shown that in a rat model of left anterior descending ligation-induced myocardial ischemia‒reperfusion injury, XBP could improve cardiomyocyte apoptosis by inhibiting autophagy and ER stress to improve cardiac function (Yang et al., 2022). The efficacy of XBP for CHF has also been demonstrated in clinical practice (Xu and Qi, 2019). However, it is disappointing that the evidence from these clinical randomized controlled trials is inadequate and shows significant methodological flaws, such as prevalent use of small samples, lack of preregistered study protocols, unreasonable random assignment methods, failure to implement blinding, and single-center studies (Wang et al., 2022).

**TABLE 1:** Components of Xinbao pill (intervention drug).

Scientific name	Chinese Pinyin	Chinese Name	Latin scientific name	Part & form used
*Moschus (the dried preputial secretion of Moschus berezovskii, M. sifanicus or M. moschiferus)*	She Xiang	麝香	Moschus	the dried preputial secretion of Moschus berezovskii
Panax ginseng C. A. Meyer	Ren Shen	人参	Panax ginseng C. A. Meyer	Dried root
*Cinnamomum verum J. Presl [Lauraceae]*	Rou Gui	肉桂	Cinnamomum cassia Presl	Dried bark
*Datura metel L. [Solanaceae]*	Yang Jin Hua	洋金花	Dature Stramonium Datura L	Dried flower
*Aconitum carmichaeli Debeaux [Ranunculaceae]*	Fu Zi	附子	Aconiti Lateralis Radix Praeparata	Root
*Panax notoginseng (Burkill) F.H.Chen [Araliaceae]*	San Qi	三七	Panax notoginseng (Burkill) F. H. Chen ex C. H.	Dried roots and rhizomes
*Bufonis Venenum*	Chan Su	蟾酥	Bufo bufo gargarizans Cantor	the dry secretion of Bufo bufo gargarizans Cantor or Bufo melanostictus Schneider
*Cervi Cornu Pantotrichum*	Lu Rong	鹿茸	Cervus nippon	the unossitized, densely hairy young horn of a buck by Cervus Nippon Temminck or Cervus elaphus Linnaeus
Cinnamomum camphora (L.) J. Presl	Bing Pian	冰片	Borneolum Syntheticum	-

The clinical efficacy and safety of XBP in the treatment of CHF is still unclear. Therefore, we conducted a multicenter, randomized, double-blind, placebo-parallel controlled trial to try to elucidate the clinical efficacy and safety of XBP in the treatment of CHF, to provide high-quality evidence-based medical evidence. The main hypothesis of this study is that XBP is superior to placebo in patients with CHF when combined with conventional therapy.

## Methods and analyses

### Design and settings

**FIGURE 1:**
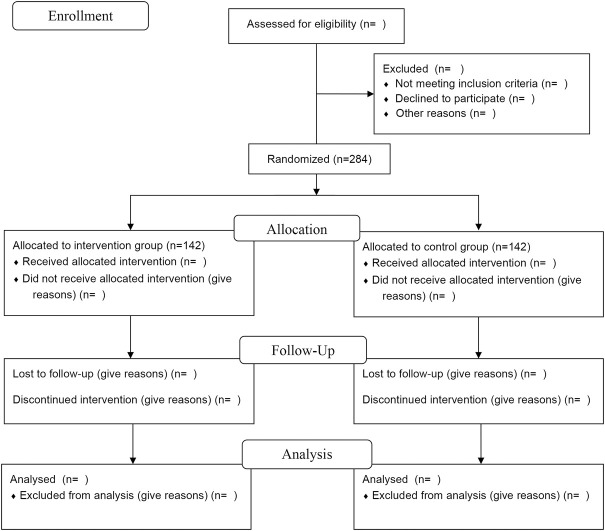
The flow diagram of this study.

This study is a multicenter, stratified, randomized, double-blind, placebo-controlled, parallel-group superiority trial planned to be implemented in patients with CHF with “heart-kidney yang deficiency syndrome.” A total of 284 participants will be recruited. After participants are enrolled and provided written informed consent, they will be randomly assigned, in a 1:1 ratio, to either the XBP group or the placebo group. The trial will consist of a 1-week screening period, 12-week intervention period, and 12-week follow-up period. The study flow is shown in Figure 1.

## Recruitment

Announcements and recruitment advertisements will be posted on WeChat, hospital outpatient waiting halls and community bulletin boards for participant recruitment. Researchers will explain the purpose of the study, the interventions, and the benefits and risks to the patients. Patients who agree to participate will be examined and diagnosed separately by two chief Chinese medicine practitioners with more than 5 years of medical experience to determine whether they meet the inclusion criteria and will be enrolled in the online allocation system after written informed consent is obtained. In addition, the demographic characteristics of ineligible patients and reasons for nonparticipation will be recorded. Enrollment begins from 1 November 2020 to 31 December 2024. Costs incurred in connection with the study will be free of charge (e.g., physical examination, laboratory test costs, *etc.*). Recruitment will be conducted at six centers nationwide, including Shunde Hospital of Guangzhou University of Chinese Medicine, Er sha Branch of Guangdong Provincial Hospital of Chinese Medicine, University City Branch of Guangdong Provincial Hospital of Chinese Medicine, Second Guangdong Provincial Hospital of Chinese Medicine, Shandong Provincial Hospital of Chinese Medicine, and Huizhou City Hospital of Chinese Medicine.

## Eligibility criteria

### Diagnostic criteria of CHF

The diagnostic criteria were referenced in the 2018 Chinese Heart Failure Diagnosis and Treatment Guidelines published by the Chinese Medical Association Cardiovascular Disease Branch (Group et al., 2018). Among them, the grading of the severity of cardiac function is based on the New York Heart Association (NYHA) cardiac function grading criteria (Yancy et al., 2013). NYHA Class I: the patient has heart disease, but physical activity is not limited. General physical activity does not cause excessive fatigue, palpitations, shortness of breath, or angina; Class II: The patient has heart disease such that physical activity is mildly restricted. No symptoms at rest, general physical activity causes excessive fatigue, palpitations, shortness of breath or angina; Class III: The patient has heart disease such that physical activity is significantly limited. No symptoms at rest, but less than general physical activity can cause excessive fatigue, palpitations, shortness of breath or angina; and Class IV: Patients with heart disease, also have symptoms of cardiac insufficiency or angina at rest, and any physical activity increasing discomfort.

### Diagnostic criteria of traditional Chinese medicine syndrome

The diagnostic criteria for “heart-kidney yang deficiency syndrome” are based on the Expert Consensus on CHF Treatment in Chinese Medicine (Alliance et al., 2014). The main symptoms include palpitations, shortness of breath, weakness, shortness of breath with movement, cold body and limbs, puffiness and swelling, abdominal distension and loose stools, dull complexion, pale tongue with thin coating, sunken and weak pulse, or shortness of breath, astringency, and knotted generation. The diagnosis of “heart-kidney yang deficiency syndrome” is based on the presence of one primary symptom and two secondary symptoms. Two experienced chief physicians confirmed the “heart-kidney yang deficiency syndrome” diagnosis.

### Inclusion criteria

Patients will be included in this study if they meet the following criteria: 1) meet the diagnostic criteria for CHF, and the Chinese medical evidence of “heart-kidney yang deficiency syndrome”; 2) 18 years old ≤ age ≤80 years old, regardless of sex; 3) have a history of CHF for more than 3 months or clinical findings of heart failure symptoms for more than 3 months; 4) NYHA heart function classification II-III, or stable clinical symptoms, including those who have been diagnosed within 2 weeks before enrollment or NYHA Class IV; 5) serum N-terminal prohormone of brain natriuretic peptide (NT-proBNP) level ≥450 pg/ml; 6) have received conventional basic treatment for at least 2 weeks and have not undergone a dose adjustment; and 7) voluntary participation, clearly understanding and signed the informed consent form.

### Exclusion criteria

Patients who meet one of the following criteria will be excluded from this study.1) heart failure due to valvular disease, congenital heart disease, pericardial disease, arrhythmia and noncardiogenic causes, or heart failure due to liver, kidney and other important organ failure; and right heart failure and acute heart failure with clear pulmonary or other causes; 2) those who plan to undergo coronary revascularization therapy or cardiac resynchronization therapy in the near future, and those who have already undergone cardiac resynchronization therapy; 3) patients with severe primary diseases of the liver, kidney and hematopoietic system, liver transaminases, alkaline phosphatase exceeding 3 times the upper limit of the normal values, blood creatinine >2 mg/dl (176.82 μmol/L), blood potassium >5.5 mmol/L; patients with tumors, severe neuroendocrine disorders and psychiatric disorders; 4) patients with left ventricular outflow tract obstruction, myocarditis, large aneurysms, sandwich aneurysms, or unrepaired heart valves causing significant hemodynamic changes; 5) patients with cardiogenic shock, uncontrollable malignant arrhythmias, progressively worsening unstable angina pectoris or acute myocardial infarction; 6) patients with uncontrolled hypertension, systolic blood pressure ≥180/mmHg and/or diastolic blood pressure ≥110 mmHg; 7) patients who are also using other drugs with cardiotonic, or diuretic effects, such as digitalis; 8) patients who are taking other Chinese herbal medicines within 1 month; 9) pregnant or preparing for pregnancy and lactation; 10) allergic patients or those with a known allergy to the treatment drugs; 11) patients with internal heat of Yin deficiency, hyperactivity of the liver and Yang, or phlegm and fire; 12) patients with glaucoma; or 13) patients who, in the judgment of the investigator, cannot complete the study or cannot comply with the requirements of the study.

### Termination criteria

The clinical trial will be terminated if any of the following occur during the course of the trial.1) discontinuation of treatment at any time; 2) an allergic reaction clearly associated with the study drug; 3) an adverse sign or symptom (for example, vomiting, diarrhea, dizziness and headache, weakness, *etc.*) clearly associated with the study drug, or an abnormal test result that, in the judgment of the investigator, warrants discontinuation of the study; 4) pregnancy in a woman; 5) an endpoint event. When a trial is discontinued, all trial records should be retained for review.

### Withdrawal criteria

All screened and qualified subjects for entry into the trial who complete the informed consent form will not be affected by their subsequent treatment, regardless of when or why they withdraw from the study. Patients have the right to withdraw from the study at any time and for any reason, but every effort should be made to avoid unnecessary patient withdrawals and to complete follow-up visits whenever possible to collect data for analysis of efficacy and safety. However, when a patient decides to withdraw, the investigator should contact the patient or their responsible relative by telephone or personal visit and confirm the reason for withdrawal if possible. The investigator should retrieve the remaining medication at the time of withdrawal, perform complete a final assessment, complete a case report if possible, explain the reason for withdrawal, and follow up on the occurrence of the endpoint event in the withdrawing patient. If the patient withdraws for reason of an adverse event, it should be documented in the complete case report form (CRF).

### Sample size

Investigators conducting the trial estimate the sample size based on the level of NT-proBNP, the main efficacy indicator. The literature (Li et al., 2013), showed that NT-proBNP levels decreased by more than 30% after 12 weeks of conventional basic treatment combined with placebo in 31.98% of patients, and NT-proBNP levels decreased by more than 30% after 12 weeks of conventional basic treatment combined with herbal medicine in 47.95% of patients. Therefore, it is expected that the percentage of patients with NT-proBNP levels decreasing more than 30% after 12 weeks of conventional basic treatment combined with XBP will be 50%. Thus, assuming *α* = 0.05 and *β* = 0.20, the calculated sample size required for each group was 114 cases after substituting the above values using PASS11 software. Considering that 20% will miss visits, the sample size was adjusted to 142 cases in each group. The total number of cases required for both groups was 284.

### Randomization and allocation

Participants will be randomly assigned, in a 1:1 ratio, to the experimental and control groups using the central randomization system of the Drug Clinical Trial Management Office, Shunde Hospital of Guangzhou University of Chinese Medicine. Randomization groups will be stratified by the central stratification method, generating random numbers, and generating randomized results by SAS statistical software (version 9.4; SAS Institute, Cary, NC, United States). The randomization number and drug number will be separately obtained by each study subcenter investigator using a sequential number in the central randomization system. All center numbers, sequence numbers, randomization numbers, and drug numbers will be managed by the statistical unit.

### Blinding

Blinding codes will be made after the randomization operation. This process will be performed by a special person, and the sequence number of the subject, the corresponding random numbers and the grouping result (i.e., subjects are assigned to either the test or control group) will be the primary blinding base; then the two groups will be blinded to the medication, which will be the secondary blinding base, and the drug number of each subject will be randomly prepared in order, and all operations will be recorded and maintained. All study personnel, participants, physicians, medication administrators, and dispensing nurses will be unaware of the group and treatment type until the study is completed. Unblinding occurred if a participant have a serious adverse event during the course of the study or require emergency care in an emergency situation. Once unblinded, the participant will be withdrawn from the study and the investigator will report the reason to the examiner within 24 h.

### Interventions

Eligible participants will be randomly assigned to either the XBP group or the placebo group. If XBP was used prior to randomization, a 2-week drug washout period was implemented to avoid any potential complications of XBP. All participants will receive conventional basic treatment.

Patients in the experimental group will receive XBP (approval number: Z44021843, 4 pills at 60 mg each, po, three times a day). Patients in the control group will receive XBP placebo (4 pills at 60 mg, po, three times a day). The appearance, taste and weight of the placebo will be the same as those of the XBP test drug. Both XBP and placebo will be manufactured by Guangdong Xinbao Pharmaceutical Technology Co., Ltd. And all drugs should meet the requirements of the Chinese National Drug Manufacturing Code. The total treatment course will be 12 weeks.

Conventional basic treatment was provided in accordance with the 2018 Chinese Heart Failure Diagnosis and Treatment Guidelines published by the Chinese Society of Cardiovascular Diseases (Group et al., 2018), including use of ACEIs, ARBs, ARNIs, diuretics, β-blockers and other drugs to reduce cardiac load and enhance myocardial contractility. Each patient should continue to receive the same type and dose of drugs in the conventional basic treatment protocol as that of before enrollment during the treatment period of the trial. No further adjustments can be made during the entire treatment period. If the condition requires a medication adjustment, it should be recorded in the case report form, and investigators should record the combined endpoints and adverse events that occur in patients who receive an increased or decreased dose of medication or type of a different medication.

Concomitant therapy for comorbidities (e.g., hypertension, angina pectoris, hyperlipidemia, and other chronic conditions) will be allowed during the trial. Investigators should accurately document concomitant medications and maintain dose stability for the duration of the trial. Patients will adhere to heart-healthy diet during the trial, such as a low-salt diet and moderate water intake, in addition to receiving counseling on appropriate lifestyle improvements such as weight monitoring, physical activity, smoking cessation, and alcohol cessation. Adherence will be evaluated by complete documentation of drug distribution and retrieval, and subjects will be judged to be following the protocol if the actual amount of drug taken is within 80%–120% of the applied drug amount.

## Outcomes

### Primary outcomes

Details of the observational items and the time window for data collection are shown in Table 2. The primary outcome will be the proportion change of patients with a decrease of more than 30% in serum NT-proBNP after treatment.

**TABLE 2:** Study procedure table.

Study phase time	Baseline period	Intervention period			Follow-up
	Visit 1	Visit 2	Visit 3	Visit 4	Visit 5
	−7 to 0 days	4 weeks	8 weeks	12 weeks	24 weeks
Data collection at baseline					
Inclusion/exclusion criteria	×				
Informed consent	×				
Demographic data	×				
Obtain the central random number	×				
Previous history, medical history, and allergies	×				
Efficiency evaluation					
NT-proBNP	×	×	×	×	×
Echocardiographic parameters	×	×	×	×	×
NYHA class	×	×	×	×	×
6MWT	×	×	×	×	×
MLHFQ score	×	×	×	×	×
TCM symptom scores	×	×	×	×	×
Clinical-endpoint events	×	×	×	×	×
Safety evaluation					
Vital signs	×	×	×	×	×
Physical examination	×	×	×	×	×
Blood routine	×	×	×	×	×
Urine routine	×	×	×	×	×
ECG	×	×	×	×	×
Blood biochemistry	×	×	×	×	×
Chest radiograph	×			×	×
Other work					
Dispense drug	×	×	×	×	
Recovery and record of study drug		×	×	×	
Record AEs		×	×	×	
Complications due to medications		×	×	×	
Evaluate compliance		×	×	×	×

NYHA, New York heart association; 6MWT, 6-min walk distance test; MLHFQ, Minnesota living with heart failure questionnaire; ECG, electrocardiogram; AE, adverse event; “x” represents the need for implementation at this point in time.

### Secondary outcome indicators

1) cardiac function classification (NYHA) (Yancy et al., 2013); 2) the 6-minute walking distance test (6MWT) (Ingle et al., 2005); 3) the Minnesota Living with Heart Failure Questionnaire score (Fitz et al., 2021); 4) the rate of endpoint events (acute exacerbation rehospitalization of CHF, cardiac death and all-cause death, *etc.*); 5) serum NT-proBNP level; 6) cardiac function indicators, such as the left ventricular ejection fraction (LVEF), the left ventricular end-diastolic diameter (LVEDD), and the left ventricular end-systolic diameter (LVESD); and 7) TCM symptom scores (Supplementary material 1) will be used to quantify the symptoms. Differences in TCM symptom scores between groups at baseline and week 4, week 8, week 12 and week 24 post-treatment will be compared and differences between groups will be calculated. The scoring process will be carried out independently by two chief TCM physicians with more than 5 years of medical experience, with any discrepancies being resolved through consultation. The comprehensive efficacy criteria were as follows: 1) ineffective: no significant improvement or even worsening of symptoms and signs, and a reduction in symptom score <30%; 2) effective: improvement of symptoms and signs, and a reduction in symptom score ≥30%; 3) significant effect: significant improvement of symptoms and signs, and a reduction of syndrome score ≥70%. TCM symptom scores = [(posttherapy score - pretherapy score)/pretherapy score]  ×  100%. Outcome indicators will be collected at week 4, 8, 12 and 24 after inclusion in the study.

### Safety outcomes

Safety outcomes include adverse event evaluation, clinical laboratory indicators (routine blood: hemoglobin, red blood cells, white blood cells, platelets; routine urine: urine protein, urine white blood cells, urine red blood cells; serum biochemistry: urea nitrogen, creatinine, ghrelin, ghrelin, fasting glucose, glycated hemoglobin, potassium, sodium, chloride) and 12-lead electrocardiogram.

### Adverse events

Adverse events will be monitored continuously during treatment and follow-up. Adverse events occur during the course of the study, especially those related to the trial drug, will be followed until the patient’s condition returns to baseline status or stabilizes. If the patient’s condition does not return to baseline or stabilize after follow-up, a note should be documented in the CRF. Any serious adverse events during the clinical trial will be reported to clinical trial supervisor, principal investigator, and drug manufacturer within 24 h. Moreover, the investigator will fill out a serious adverse reaction form to record the time of occurrence, severity, duration, measures taken and regression of the serious adverse event. The investigator will also make a judgment on whether the abnormal laboratory results are clinically significant and provide possible explanations. Abnormal laboratory results resulting from an adverse event that has already been reported should also be recorded as an adverse event. Clinically significant abnormal laboratory tests that meet one or more of the following conditions should be recorded as a separate diagnosis on the adverse events page of the CRF (excluding abnormal laboratory results resulting from an adverse event that has already been reported): 1) if accompanied by clinical symptoms; 2) if a change in the study medication has occurred; or 3) if a change in the combination medication and/or other therapeutic measures is required.

### Data collection and management

Study data will be recorded on the paper CRF or electronically *via* the eCRF. The paper CRFs will be sent to the clinical center and Shunde Hospital of Guangzhou University of Chinese Medicine will provide training and support to the investigators by using the eCRF system. To promote participant retention and completion of follow-up visits, frontline investigators will inform patients the time of follow-up visit to hospital by phone and WeChat. This work will be performed by two independent investigators.

The data management process needs to comply with regulatory requirements such as “Quality Management Standards for Drug Clinical Trials” (Zhang et al., 2021) and “Technical Guidelines for Clinical Trial Data Management Work” (Wang et al., 2013)to ensure the traceability of clinical trial data. The data manager will design the database based on the CRF using Epidata software and will release it after testing. The clinical research coordinator (CRC) is responsible for entering the data on the CRF into the database, and the data entry is performed in a secondary manner. The data manager will prepare the data verification SAS program based on the data verification plan and then generate a data query form, which is returned to the data manager by the clinical reviewer after the investigator has answered the queries, and the data manager will revise the database accordingly. MedDRA 21.0 or later will be used for adverse event coding. After database cleanup is completed, the data manager will write the data verification report for use in data verification meetings. The database lock list is completed at regular intervals and the database lock is completed according to the database lock procedure. After the database lock is completed, the data manager will export the data file in SAS format and send it to the statistician for statistical analysis. The personal information about of the potential and enrolled participants is confidential and will be protected throughout the trial, and researchers will be expected to maintain data confidentiality for 5 years after trial termination.

## Statistical analyses

### Statistical analysis data sets

Subjects in the trial will be divided into full analysis set (FAS), per-protocol analysis (PPS), and a safety analysis set (SAS). Efficacy analysis will be performed on the FAS and PPS. All baseline demographics will be analyzed on the FAS, and safety evaluation will be performed on the SAS.

### Statistical analysis methods

All statistical tests will be performed using a two-sided test, and a *p* value less than or equal to 0.05 will be considered statistically significant for the difference tested. Dichotomous indicators will be used to describe the number of cases and percentages of each category. Quantitative indicators conforming to normal distribution will be expressed as the “mean ± standard deviation” and skewed data will be described as the median, lower quartile (Q1), and upper quartile (Q3). Comparisons between the two general groups will be analyzed using appropriate methods depending on the type of indicator. Paired *t* test or Wilcoxon rank sum test will be used for comparison between groups for quantitative data, chi-square test or Fisher exact probability method for categorical data, and Wilcoxon rank sum test or CMH test for rank data. The specific applicable statistical methods for each outcome indicator are shown in Table 3.

**TABLE 3:** Outcomes and methods of analyses.

Outcome/variable	Hypothesis	Measures	Methods of analyses
Baseline balance test		Quantitative outcomes (age, temperature, heart rate, respiratory rate, and blood pressure)	*t*-test/Wilcoxon rank-sum test
		Qualitative outcomes (sex, marriage, and previous treatment)	Chi-squared test/Fisher’s exact test/rank-sum test
Primary outcome			
Percent and cases of NT-proBNP decreased ≥30%	decrease occurred		Chi-square test/Fisher’s exact test
Secondary outcome			
NYHA class	decrease occurred		Wilcoxon rank-sum test
6MWT	increase occurred		*t*-test/Wilcoxon rank-sum test
MLHFQ score	decrease occurred	Questionnaire	*t*-test/Wilcoxon rank-sum test
Clinical-endpoint events	decrease occurred	Clinical-endpoint event rate	Log-rank test
NT-proBNP	decrease occurred		*t*-test/Wilcoxon rank-sum test
Echocardiographic parameters	Improvement occurred	LVEF, LVEDD, LVESD	*t*-test/Wilcoxon rank-sum test
TCM symptom scores	decrease occurred	Disappearing rate of main symptoms	Chi-square test/Fisher’s exact test
Safety outcomes			
AEs, SAE		Percent and cases of AEs and SAEs	Chi-square test/Fisher’s exact test

NYHA, New York heart association; 6MWT, 6-min walk distance; MLHFQ, Minnesota living with heart failure questionnaire; LVEF, left ventricular ejection fraction; LVEDD, left ventricular end-diastolic diameter; LVESD, left ventricular end-systolic diameter; AE, adverse event; SAE, serious adverse events; TCM, traditional Chinese medicine.

### Medication adherence analysis

Pearson’s chi-square test or Fisher’s exact chi-square test will be used to compare whether the patients in both groups are using the trial medication at the right time and at the right dosage but not using the medication prohibited in the protocol. The number of combined medication users in each group should also be counted and tabulated in detail.

### Quality control

To ensure the quality of this trial, each study center will have a study leader and several fixed study components. The requirements of the clinical study protocol will be strictly followed. The technical staff in the group leader unit will maintain close contact with each study center at all times and will visit each study center in the early, middle, and late stages of the study to check the case observation records and solve any problems that may arise in a timely manner. Each party will conduct the clinical study in strict accordance with the approved protocol, and any deviation from the protocol will be recorded. Any modifications to the study protocol need to be reported to the ethics committee for approval before implementation. Meanwhile, a steering committee and data monitoring committee composed of experienced clinical and academic researchers in the field has been developed at Shunde Hospital of Guangzhou University of Chinese Medicine, and the committee will conduct monthly online audits and half-yearly on-site audits.

### Trial status

This is an ongoing trial. The first participant was recruited on 3 December 2021. This study is currently recruiting participants.

## Discussion

XBP, as an adjuvant treatment for CHF, has been widely used in China, however, its clinical efficacy and safety are still unclear due to the lack of support provided by high-quality clinical trial (Wang et al., 2022). This trial is the first multicenter, stratified, randomized, double-blind, placebo-controlled, parallel-group superiority trial conducted in China to investigate the safety and clinical efficacy of XBP as an adjuvant therapy for CHF and to provide high-quality evidence for XBP in the treatment of CHF.

Compounded Chinese medicine is the most prominent form of TCM used to treat diseases (Tang et al., 2018; Luan et al., 2020). However, researchers experience challenges in standardizing clinical randomized controlled trials on compound Chinese medicine (Lin et al., 2018). First, it is difficult to conduct clinical studies under the guidance of the “syndrome differentiation and treatment” theory of TCM(Jiang et al., 2012). The principle of TCM compounding is to treat patients based on different clinical manifestations of diseases under the guidance of TCM theory, which is summarized as “syndrome,” and then to administer the corresponding Chinese medicine treatment (Hu et al., 2022). According to the theory of TCM, XBP mainly treats CHF patients with “heart-kidney yang deficiency syndrome.” However, in actual clinical practice, patients present with different symptoms so the treatment approach is subjective based on the doctor’s belief, and the same patient may even receive a different diagnosis from a different doctor (Hao et al., 1990). This challenge has limited the development of clinical research on XBP, so previous clinical studies has not been often focused on the clinical efficacy and safety of XBP in the treatment of heart failure patients with “heart-kidney yang deficiency syndrome.”

To avoid including patients with other “syndromes” and to clarify the diagnosis of “heart-kidney yang deficiency syndrome,” the recruited patients were cross-checked by two chief TCM physicians with more than 5 years of medical experience. Second, because XBP has a unique color, smell and taste as those of a natural drug, it is difficult to simulate it with ingredients that have no therapeutic effect, which makes the preparation of placebo more difficult (Shi and Yuan, 2022). Preparation of placebo is critical in aspect of implementing double-blindness, which can effectively avoid bias, and the level of placebo preparation determines the chance of breaking the blindness of the subjects and investigators during the implementation of the blinding method (Kaptchuk, 1998). The XBP and placebos used in this study were manufactured by Guangdong Xinbao Pharmaceutical Co., Ltd. to ensure that placebos were as consistent as possible with the test drug in terms of appearance, color, odor, and taste.

The NT-proBNP level is recommended by guidelines as the most valuable and reliable biomarker for the diagnosis of HF(Ponikowski et al., 2016), and is widely used for differential diagnosis, risk stratification and prognostic assessment of CHF. Studies have shown that the risk of CHF-related death is positively correlated with NT-proBNP levels (Cao et al., 2019), so the proportion of patients with a decrease in serum NT-proBNP of more than 30% was chosen as the primary outcome for this study. A meta-analysis showed strong evidence that the 6MWT is moderately to highly relevant in evaluating the efficacy of cardiac rehabilitation for patients (Bellet et al., 2012), so the study will use the 6MWT as a secondary outcome. Moreover, the secondary outcome in terms of TCM symptom score, quantified symptoms to reflect objectivity, and perfectly combined TCM evidence with modern medicine.

Several previous prospective clinical studies (Wang et al., 2019; Zhao et al., 2019; Lu et al., 2020) have shown that combining XBP therapy with conventional therapy appears to be beneficial in improving cardiac function (e.g., increasing LVEF, and decreasing LVEDD and LVESD), decreasing BNP levels, and increasing 6MWT in patients with CHF. However, it is important to note that all of these clinical studies published in Chinese journal articles had serious methodological design flaws (Wang et al., 2022). First, none of the study protocols were preregistered prior to the initiation of the study, which would have improved transparency and avoided bias in selective outcome reporting and publication bias. Second, all were small sample (sample size ≤120 cases), single-center studies. In addition, no placebo-controlled trials were planned to exclude placebo effects. Finally, studies based on non-double-blinded design reduced the authenticity and reliability of data collection and result evaluation. Due to these shortcomings, reliable evidence on the clinical use of XBP in patients with CHF is still lacking. In our ongoing clinical study, we will thoroughly address these methodological shortcomings and will provide high evidence-based medical proof for the use of XBP as a complementary alternative therapy for patients with CHF.

However, the present study protocol also has limitations that are worth discussing. First, because the study period was limited, the follow-up period of this trial was relatively short. Although XBP is effective in reducing overall mortality, long-term prognosis can not be evaluated. Along-term follow-up is needed to obtain additional data. Secondly, our inclusion population is restricted to adult heart failure patients under 80 years of age, and it is unclear whether this is applicable to older people over 80 years of age. Thirdly, although our study is a multicentre study, the main study population is predominantly Han Chinese in southern China and it is unknown whether it is applicable to other ethnic and regional populations. Finally, researchers in this trial only investigated the adjuvant effect of XBP on conventional basic treatment, and its individual therapeutic effect is still unclear.

### Patient and public involvement

Patients and/or the public were involved in the design, conducting, reporting, dissemination plans of this research. Refer to the Methods section for further details.

## Abbreviations

ACEI, angiotensin-converting enzyme inhibitor; ARB, Angiotensin receptor blocker; ARNI, angiotensin receptor neprilysin inhibitor; CHF, chronic heart failure; CRF, case report form; CRC, clinical research coordinator; eCRF, electronic case report form; FAS, full analysis set; MRA, mineralocorticoid receptor antagonists; NT-proBNP, N-terminal prohormone of brain natriuretic peptide; NYHA, New York Heart Association; PPS, per-protocol analysis; SAS, safety analysis set; TCM, traditional Chinese medicine; XBP, Xinbao pill.
